# Pregnancies in Women with a Previous Complete Uterine Rupture

**DOI:** 10.1155/2023/9056489

**Published:** 2023-02-09

**Authors:** Iqbal Al-Zirqi, Siri Vangen

**Affiliations:** ^1^Norwegian Research Center for Women's Health, Oslo, Norway; ^2^Division of Gynaecology and Obstetrics, Rikshospitalet, Oslo University Hospital, Oslo, Norway; ^3^Institute of Clinical Medicine, University of Oslo, Oslo, Norway

## Abstract

**Objective:**

To study the outcomes of new pregnancies after a previous complete uterine rupture.

**Design:**

Descriptive study based on population data from the Medical Birth Registry of Norway, the Patient Administration System, and the medical records. *Sample*. Maternities with a previous complete uterine rupture in Norway during the period 1967–2011 (*N* = 72), extracted from 2 455 797 maternities.

**Method:**

We measured the rate of new complete ruptures and partial ruptures, as well as the maternal and perinatal outcomes of these pregnancies. The characteristics of both previous ruptures and new ruptures were described.

**Results:**

Among 72 maternities, there were thirty-seven with previous ruptures in the lower segment (LS) and 35 outside the LS. We found three new complete ruptures and six uneventful partial ruptures, resulting in a rate of 4.2% and 8.3%, respectively. All three complete ruptures occurred preterm in scars outside the LS. The rate of the new complete rupture was 0% in those with previous ruptures in the LS and 8.6% in those with previous ruptures outside the LS. The corrected perinatal mortality was 1.3%, and prematurity (<37 weeks) was high (36.1%); this was noticed even in the absence of new ruptures and was mostly iatrogenic. Two hysterectomies were performed in the absence of rupture and two cases had abnormal invasive placenta.

**Conclusion:**

The prognosis for pregnancies after a previous complete uterine rupture is favorable. Prematurity is a problem caused by both obstetrician and mother's anxiety; therefore, the timing of delivery is the most challenging. Management should include careful counseling, vigilance for symptoms, and immediate delivery when a rupture is suspected.

## 1. Introduction

The rate of uterine rupture is increasing worldwide in relation to increased use of caesarean section (CS) [1]. As a result, an increasing number of women who have experienced a complete uterine rupture are asking for advice regarding a new pregnancy. Complete uterine rupture, which is often catastrophic, involves all uterine wall layers, including the serosa and membranes [2, 3]. Much more common is the uneventful partial rupture (dehiscence), which spares the serosa or membranes. In earlier times, hysterectomy was performed in the event of a complete rupture based on the assumption that the uterine wall is so weak that it cannot tolerate a new pregnancy. In 1969, Reyes-Ceja et al. [4] found only one new rupture among 22 pregnancies with previous ruptures. Based on this finding, the surgical repair of uterine rupture was recommended instead of hysterectomy.

Very few studies have investigated the risk of repeat rupture during pregnancy. Most publications are case reports, in which the rate of repeat rupture varies between 0 and 33% [5]. However, a recent research has indicated a rate of repeat rupture of 4 to 13.7%. [6]. Current guidelines from the American College of Obstetricians and Gynecologists (ACOG) recommend that elective repeat CS should be scheduled between 36 and 38/6/7 weeks of gestation for pregnant women with a history of uterine rupture, with eventual changes based on individual evaluation (ACOG 2017) [7].

The aim of the present study was to determine the rate of repeat complete rupture in new pregnancies and the outcomes of such pregnancies. Describing the outcome of these pregnancies may contribute to the individual evaluation of each woman regarding advice on a new pregnancy, follow-up, and timing of delivery.

## 2. Materials and Methods

### 2.1. Design and Study Population

This was a descriptive, retrospective, population-based study looking at the outcomes of new pregnancies in mothers with previous complete uterine rupture. Data were extracted from all births in Norway registered from 1967 to 2011 (*N* = 2455797). Two registries were used: the Medical Birth Registry of Norway (MBRN) and the Patient Administration System (PAS). The MBRN contains information on all births in Norway after 16 weeks of gestation. Midwives attending a birth fill out and send a standardized MBRN form within 7 days after delivery. The PAS is a local registry at each maternity unit that maintains records of all inpatient diagnoses. All ruptures were identified using international diagnostic codes. In the MBRN, the internal code for uterine rupture was 71 prior to 1999; from 1999 to the present, the diagnostic codes are O710 and O711 based on the 10th revision of the international classification of diseases (ICDs) [8]. In the PAS, the uterine rupture was identified by the ICD8 code (1967–1978) [9], ICD9 codes 6650 and 6651 (1979–1998) [10], and ICD10 codes O710 and O711 (1999–present) [8]. These codes do not specify the rupture type (complete or partial), which was, however, identified in the medical records. Complete rupture was defined as the rupture of all uterine wall layers, including the serosa and amniotic membranes, and partial rupture as rupture of the uterine wall, sparing the serosa or membranes. All births associated with a uterine rupture were identified by the first author, who visited maternity units in Norway and reviewed maternal medical records. Those with complete uterine ruptures were followed (*n* = 274).

The study population was mothers with new pregnancies and childbirths after a previous complete uterine rupture (*n* = 72). Mothers did not have any operative procedure in the interval between rupture repair and the new pregnancy. We studied the characteristics of previous complete ruptures and the rate of repeat ruptures and the outcomes of these pregnancies.

The Regional Ethics Committee (2010/1609–4) and the Data Inspectorate of Norway approved the study. The South-Eastern Norway Regional Health Authority funded the study of the uterine rupture as a post-PhD project for the lead author.

A preprint has previously been published [11].

## 3. Variables

### 3.1. Outcome Measures

The outcome measures were maternal and infant outcomes, mode of delivery, and gestational age at the delivery of the new pregnancies. Maternal outcomes were recorded as uncomplicated, repeat complete rupture, partial rupture, peripartum hysterectomy, and abnormally invasive placenta. Infant outcomes were recorded as healthy, moderate asphyxia, severe asphyxia, admission to neonatal intensive care unit (NICU), and perinatal death (stillbirth and neonatal death). All were categorized as either Yes or No.

The mode of delivery was grouped into elective CS, emergency-prelabour CS, and vaginal delivery. Gestational age at delivery was grouped into weeks: <28, 28–32, 33–36, 37–38, and 39 weeks.

Peripartum hysterectomy was defined as the surgical removal of the uterus at the time of delivery or up to 42 days postpartum, excluding hysterectomy because of cancer. Abnormally invasive placenta included all types of morbidly adherent placenta defined by histology, including accreta, increta, and percreta.

Stillbirth was defined as infant death before birth caused by asphyxia related to uterine rupture, and neonatal death was defined as death ≤7 days after birth. We defined severe asphyxia [8] using diagnostic ICD-10 code P21.0 (asphyxia with 1-minute Apgar score 0–3). We defined moderate asphyxia [8] using ICD-10 code P21.1 (asphyxia with 1-minute Apgar score 4–7). NICU admission was defined as admission due to prematurity. A healthy infant was defined as one without any complications and no admission to the NICU.

### 3.2. Characteristics of Previous Ruptures

Characteristics of previous ruptures included place of rupture (within lower uterine segment [LS; reference] and outside LS), gestational age at rupture (<37 weeks and ≥37 weeks [reference]), time of occurrence of rupture (before labour start and after labour start [reference]), detection time (during CS or postpartum after vaginal delivery/CS), and presence of perinatal death. In addition, the interdelivery interval was included and defined as the time from complete rupture to new delivery (1 year, 2–3 years [reference], and ≥4 years).

### 3.3. Data Analysis

We used frequency analysis to calculate the rates and outcomes of a new complete and partial uterine rupture. Cross tabulation with odds ratios (ORs) and 95% confidence intervals (CIs) was used to measure the risk of new rupture based on different characteristics of previous ruptures. We used SPPS 26 for statistical analyses.

## 4. Results

A total of 274 women had previous complete uterine ruptures. We studied 195 women after excluding 3 maternal deaths, 56 hysterectomies, and 20 sterilizations. Among these women, 88 women became pregnant, 16 of whom had miscarriages (18.2%) and 72 continued the pregnancy to delivery after 28 weeks (81.8%).

Among the 72 women, 37 (51.4%) had their previous ruptures within the LS and 35 (48.6%) had their ruptures outside the LS (Table 1). Some of the previous ruptures included ruptures of scars that were not due to CS. These included three bicornuate uterus ruptures, two traumatic ruptures after traffic accident, one hysterotomy rupture at 20 weeks for termination of pregnancy, one myomectomy scar rupture, one rupture after perforation during transcervical resection of myoma, and one rupture at tubouterine area related to previous ectopic pregnancy. The previous ruptures that were outside LS included mostly ruptures in vertical scars from classical CS and ruptures in the lateral side of uterine wall; this was followed by ruptures in the posterior wall of uterine corpus as well as uterine fundus. Nearly half of the previous ruptures occurred at 37–40 weeks (47.2%); only 19.4% occurred before the start of labour. Mothers who were <35-years-old accounted for 61.1% of the study cohort. Previous ruptures resulted in 34 perinatal deaths (47.2%). The last period (2000–2011) had the highest percentage of new pregnancies.

Table 2 shows the outcomes of new pregnancies among mothers with previous complete uterine ruptures. The majority delivered by elective CS (79.2%), whereas only two women delivered vaginally. The two vaginal deliveries occurred in the first period (1967–1977); one was spontaneous and uneventful premature delivery at 32 weeks following a previous uterine rupture in the LS, while the second was a spontaneous premature twin delivery at 36 weeks following a previous traumatic rupture in the uterine fundus at 28 weeks. No rupture occurred in these two cases, but the twin delivery ended with hysterectomy due to severe atonic postpartum haemorrhage.

Three new complete ruptures occurred, resulting in a rate of 4.2%. This is significantly higher than the rate of 0.16% in new pregnancies after previous CS without a previous rupture (OR: 26.4; 95% CI: 5.3–81.5). The complete ruptures presented with acute abdominal pain, occurring at 29, 31, and 32 weeks in each of the three cases. There were six uneventful partial ruptures (8.3%) and two hysterectomies without the presence of uterine rupture (2.8%). The two hysterectomies included one case of severe postpartum haemorrhage after vaginal delivery as described above and another case with placenta accreta after four preterm CSs. No maternal deaths were recorded among the 72 mothers, and the corrected perinatal mortality was 1.3%.

Delivery at 37–38 weeks occurred in 61.1% of pregnancies, and 36.1% experienced premature delivery. This is significantly higher than the rate of 9.6% among mothers with previous CS without a previous rupture (OR: 5.3; 95% CI: 3.2–8.8).

Seven (9.7%) preterm deliveries occurred at 28–32 weeks. These included the three complete ruptures and two partial ruptures at 28 weeks. The remaining two included the previously described spontaneous premature vaginal delivery at 32 weeks and another emergency CS at 28 weeks due to pain, but no rupture was found; the previous rupture was in the LS at 37 weeks and resulted in perinatal death. There were 19 deliveries at 33–36 weeks without new ruptures, including 12 elective and 7 emergency CSs. The elective CSs included four at 36 weeks (two with twins and two with previous ruptures at 37 weeks and perinatal deaths). All four previous ruptures were in the LS. In addition, six mothers delivered electively at 35 weeks, all with previous ruptures resulting in perinatal deaths; the first one had a previous rupture in the LS after trial of labour (TOL) at 39 weeks, the second had a previous rupture at 34 weeks after TOL, the third had a large rupture outside the LS after vacuum extraction in the unscarred uterus, the fourth had a previous rupture and placenta percreta at 24 weeks, the fifth had a previous rupture outside the LS at 39 weeks in an unscarred uterus after TOL, and the sixth had bicornuate uterus and rupture in one corneum after TOL at 37 weeks. There was one elective CS at 34 weeks due to an ultrasound finding of a very thin lower segment, and one at 33 weeks due to previous prelabour rupture in the LS at 33 weeks; both previous ruptures resulted in perinatal deaths.

The seven emergency CSs at 33–36 weeks were performed due to pain and suspected rupture, or premature labour, or preeclampsia (PE); they included five at 36 weeks and two at 34 weeks. All previous ruptures were in the LS except for one with previous rupture in the accessory horn.

Only 5 of the 19 who delivered between 33 and 36 weeks had a previous rupture outside the LS (26.3%), whereas 13 (68.4%) had perinatal deaths as a result of their previous ruptures.

There were other women who had their planned CS at 38 weeks despite having a previous rupture outside the LS, or prelabour or at early gestational age (data not shown).

Table 3 shows that all three complete ruptures occurred in mothers who had previous ruptures outside the LS; the rate of repeat ruptures among those with previous rupture outside the LS was 8.6%. The partial rupture rate in this group was 11.4% compared to the rate of 5.4% for those with previous ruptures in the LS. The difference was not significant (OR: 2.3; 95% CI: 0.3–26.3). Mothers with an interdelivery interval of 2–3 years did not develop repeat complete ruptures. There was a tendency toward an increased rupture rate when the interdelivery interval was 1 year or ≥4 years vs. 2–3 years, but the difference was not significant.

There was a tendency toward an increased rupture rate when previous rupture occurred before labour start or at gestational age <37 weeks, but this also did not reach significance.

The details of the three repeat complete ruptures are provided in Table S1A. All had immediate CS upon arrival to the hospital. They resulted in one stillbirth due to asphyxia as a result of rupture, early neonatal death due to severe multiple congenital malformations without asphyxia, and one infant with moderate asphyxia.

The details of mothers who developed partial ruptures are provided in Table S1B. Five presented with mild abdominal pains or irregular contractions, whereas one was discovered coincidentally at elective CS.

Two mothers had abnormally invasive placenta in their new pregnancies (2.8%). One of them ended in hysterectomy as described above; the second one (placenta increta) was detected a complete rupture at 32 weeks, as shown in Table S1A (Case 3).

## 5. Discussion

### 5.1. Main Findings

There were three new complete ruptures among 72 mothers with a previous complete uterine rupture (a rate of 4.2%; 8.6% if the previous rupture is outside the LS and 0% if the previous rupture is in the LS). Mothers with repeat complete ruptures presented preterm with acute abdominal pain. There were six partial ruptures that were uneventful except for two premature births at 28 weeks. The corrected perinatal mortality was 1.3% and prematurity (<37 weeks) was very high (36.1%); this was noted even in the absence of new ruptures and was mostly iatrogenic. Those with an interdelivery interval of 2–3 years had zero repeat complete ruptures.

### 5.2. Strengths and Weakness

This study is the largest to date on pregnancies after a previous rupture among the whole population of a single country. Previous studies were mainly collections of reports from different countries. Our study included pregnancies after all types of previous ruptures, making the results more comprehensive. However, the data covered several periods of time, and therefore, different obstetric practices. This may have affected the rate and outcomes of repeat rupture. Because of the rarity of women becoming pregnant after uterine ruptures, we collected cases covering 44 years. Nonetheless, cases were still few, allowing only for descriptive statistical analysis.

### 5.3. Interpretation

The rate of repeat complete uterine rupture in our study was similar to the rate of 4.8% in the review by Lim from 1971 to 2005 [12]. They included five earlier studies from Ireland, the United States, Saudi Arabia, Qatar, and Nigeria, as well as five women from their unit in the Netherlands, for a total of 85 pregnancies. Half of these women were recruited from Nigeria, where all of the repeat ruptures occurred, thus giving a rate of 9% for this country.

On the other hand, a 2018 review by Frank [6] found an overall rate of 12.3% after including 11 studies from different countries with low and high resources in 1981–2015. The review found a wide range in the rate between different studies (0 to 37.5%); a study from Israel [13] in 2015 showed a recurrent rupture rate of 15.2% among 46 pregnancies. Moreover, 8.7% of pregnancies developed partial ruptures (dehiscence), similar to our rate.

The outcomes from the Israeli study occurred despite 71.7% of deliveries occurring before 37 weeks. We had a lower rupture rate despite only 36.1% of infants being delivered before 37 weeks. Moreover, we showed that timing the delivery at an earlier gestational age was not necessarily associated with a reduced risk of rupture, as all repeat complete ruptures occurred preterm. A similar finding was reported by Usta et al. [5] in a study from Beirut that included 24 pregnancies. The latter study showed no significant difference in the mean or median gestational age between pregnancies in which rupture occurred and those in which it did not. However, Ritchie [14] found that 85% of the repeat ruptures occurred after 36 weeks.

The absence of maternal deaths and the low perinatal death rate in our study reflect the importance of immediate access to emergency obstetric care, regardless of the timing of delivery.

All three repeat complete ruptures occurred in those who had a previous rupture outside the LS. This indicates that women with a previous rupture in the LS have a better prognosis as demonstrated earlier in our study on prelabour uterine ruptures [15]. However, the majority of those with previous ruptures outside the LS did not have a repeat rupture. This is in contrast to the findings of Usta et al. [5] who found 100% repeated rupture among those with previous rupture outside the LS. Our finding of zero complete ruptures when the interdelivery interval was 2–3 years is in agreement with the previous studies [2, 16]. Furthermore, we found an increased repeat rupture rate when the interdelivery interval was either 1 year or ≥4 years. This is not in agreement with Usta et al. [5] who found that the median interdelivery interval in those who had repeat ruptures was significantly shorter, 2 vs. 5 years. One should take into consideration that the sample in Usta et al. was smaller than our sample. Similar to the results reported by Usta, we found a tendency toward increased repeat rupture when a previous rupture was at gestational age <37 weeks. Our findings suggest that women with previous ruptures at an earlier gestational age, with too short an interdelivery interval or previous rupture outside the LS, should not be excluded from trying a new pregnancy. However, as there is an increased tendency towards rupture, even though it was not statistically significant, careful counseling and monitoring is important.

### 5.4. Will Our Results Help in Counseling Mothers with Previous Uterine Ruptures?

As the outcomes of new pregnancies were favorable, this may reassure women with previous ruptures, but the most challenging issue in counseling and planning is the timing of the CS. This study included different periods of time with different management approaches. In addition, there was no written consensus in the previous years on the optimal timing of delivery in our national guidelines. Therefore, the data are not appropriate for answering this question. One can see that the timing of delivery was evaluated on an individual basis and mostly affected by the presence or absence of perinatal deaths at previous ruptures. This factor increased fear among both obstetricians and mothers, leading to iatrogenic premature delivery. These data may guide us to better and more objective timing of delivery. There must be a balance between the risk of prematurity and risk of new complete uterine rupture. Admission in the last weeks, close to 37–38 weeks, may be a solution. In the Netherlands [12], there was assessment of lung maturity or administration of corticosteroid if CS was planned before 37 weeks. There was no administration of corticosteroids in our women who delivered by planned CS from 34 weeks. This might be something to consider in the future, especially in those less than 36 weeks, reducing complication of respiratory distress.

Our study showed that the risk for women with previous rupture was not only of repeat complete rupture, but also of abnormally invasive placenta (placenta accreta spectrum) and hysterectomy. The placenta accrete spectrum rate in our study (2.8%) was slightly higher than the placenta accrete rate of 2.3% after previous four CSs [17]. One cannot conclude that the previous uterine rupture by itself increases the risk of placenta accrete spectrum as there were pre-existing risk factors, such as multiple scars and previous preterm CSs. Placenta accrete spectrum is known to increase with increasing number of scars [18] or CS-performed preterm [19]. Women with previous ruptures should be counseled about this, especially if their rupture occurred at an earlier gestational age or they have multiple uterine scars. An ultrasound scoring of placenta accrete spectrum during pregnancy is suggested to predict the risk of massive intraoperative bleeding in these patients [20]; this can result in using better prophylactic procedures to reduce such bleeding [21].

No perinatal death occurred in the last period of the study, indicating a better prognosis for mothers with a previous rupture. A study regarding the outcome of pregnancy after a previous uterine rupture in the recent years would be worthwhile.

## 6. Conclusion

The prognosis for pregnancies after a previous complete uterine rupture is favorable. Prematurity is mostly iatrogenic, caused by both obstetrician and mother's anxiety; therefore, the timing of delivery is the most challenging aspect. Careful counseling of the mothers, vigilance for symptoms, and quick access to a tertiary unit are the most important points in management.

## Figures and Tables

**Table 1:** Characteristics of previous complete ruptures (*n* = 72).

	No.	(%)
Gestational age at rupture		
20–24 weeks	3	4.2
25–30 weeks	5	6.9
31–36 weeks	9	12.5
37–40 weeks	34	47.2
≥41 weeks	21	29.2
Place of rupture		
Lower segment	37	51.4
Outside lower segment	35	48.6
Vertical scar	9	12.5
Posterior wall	7	9.7
Fundus	6	8.3
Anterior wall	3	4.2
Anterior and posterior wall	2	2.8
Tubouterine angle/corneum/lateral	8	11.1
Time of occurrence		
After labour start	58	88.6
Before labour start	14	19.4
Perinatal deaths	34	47.2
Stillbirth (excluding antepartum)	19	26.4
Neonatal deaths	15	20.8

**Table 2:** New pregnancy outcomes among mothers with a previous uterine rupture (*n* = 72).

	No.	
Mode of delivery		
Elective CS	57	79.2
Emergency prelabour CS	13	18.1
Vaginal delivery	2	2.8
Gestational age at delivery		
<28 weeks	0	0.0
28–32 weeks	7	9.7
33–36 weeks	19	26.4
37–38 weeks	44	61.1
39 weeks	2	2.8
Maternal outcome		
Uncomplicated	60	83.3
Complete rupture	3	4.2
Partial rupture	6	8.3
Hysterectomy^1^	2	2.8
Abnormally invasive placenta^2^	2	2.8
Infant outcome		
Healthy	63	87.5
Moderate asphyxia	1	1.3
NICU admission^3^	6	8.3
Stillbirth	1	1.3
Neonatal death	1	1.3
PND^4^	2	2.7
PND corrected^5^	1	1.3

^1^No uterine rupture. ^2^Including one of two hysterectomies. ^3^Neonatal intensive care unit admissions due to prematurity. ^4^Perinatal death, summing stillbirths and neonatal deaths. ^5^Excluding deaths due to congenital malformations.

**Table 3:** New pregnancy outcomes based on obstetric history (*n* = 72).

	Complete uterine rupture	Partial uterine rupture
Place of previous rupture		
In lower segment (*n* = 37)	0 (0.0%)	2 (5.4%)
Outside lower segment (*n* = 35)	3 (8.5%)	4 (11.4%)
Interdelivery interval		
1 year (*n* = 16)	1 (6.3%)	2 (12.5%)
2–3 years (*n* = 36)	0 (0.0%)	2 (5.6%)
≥4 years (*n* = 20)	2 (10.0%)	2 (10.0%)
Previous gestational age		
≥37 weeks (*n* = 55)	2 (3.6%)	4 (7.3%)
<37 weeks (*n* = 17)	1 (5.9%)	2 (11.8%)
Occurrence of previous rupture		
After labour start (*n* = 58)	2 (3.4%)	4 (6.9%)
Before labour start (*n* = 14)	1 (7.1%)	2 (14.3%)

## Data Availability

The data are from the Medical Birth Registry of Norway, the Patient Administration system, and the Medical records.

## Supplementary Materials

Supplementary Materials Table S1A: mothers with new complete uterine rupture. Table S2B: mothers with new partial uterine rupture.
