# Supplementary material for: Pregnancies in Women with a Previous Complete Uterine Rupture
Source: Obstet Gynecol Int. 2023 Feb 9;2023:9056489. doi: 10.1155/2023/9056489 (PMC9935781; doi:10.1155/2023/9056489)
Supplement: Supplementary Materials — Table S1A: mothers with new complete uterine rupture. Table S2B: mothers with new partial uterine rupture. [file 9056489.f1.docx]

Table S1A. Mothers with new complete uterine rupture

|  | Obstetric history | Current delivery | Perinatal outcome |
| --- | --- | --- | --- |
| **Case 1**  (1967-1977) | Para 1  -Hysterotomy (20 weeks) with vertical incision.  -Complete rupture of vertical scar (40 weeks) after vaginal delivery (stillbirth). | -Four years after previous rupture.  -Acute abdomen (29 weeks); immediate CS: complete rupture in vertical scar; placental separation and infant extrusion. | Stillbirth due to rupture. |
| **Case 2**  (1978-1988) | Para 3  -Vaginal delivery, then emergency CS with vertical incision (breech at term).  -Complete rupture (39 weeks) in vertical scar after labour start (stillbirth). | -Five years after previous rupture.  - Acute abdomen (30 weeks); immediate CS: complete rupture in vertical scar. | Good Apgar score with no signs of asphyxia; early neonatal death due to severe multiple congenital malformations. |
| **Case 3**  (2000-2011) | Para 1  -Uncompleted transcervical resection of myoma (fundal perforation).  -Pre-labour fundal rupture  (27 weeks; neonatal death). | -One year after previous rupture.  -Mild abdominal pain/admission (25-29 weeks).  -Acute abdomen (32 weeks); immediate CS: fundal rupture & placenta increta. | Only moderate asphyxia; admitted to neonatal intensive care unit (NICU) and discharged healthy. |

Table S1B. Mothers with new partial ruptures

|  | Obstetric history | Current delivery | Perinatal outcome |
| --- | --- | --- | --- |
| Case 1 (1967-1977) | Para 2  -Emergency CS with vertical incision (term).  -Complete rupture of vertical scar (40 weeks) after labour detected at CS (healthy infant). | -Six years after previous rupture.  -Irregular contractions (38 weeks); immediate CS: partial rupture in vertical scar. | Alive and healthy. |
| Case 2 (1967-1977) | Para 1  -Traumatic complete rupture (28 weeks) in uterine fundus (traffic collision) (stillbirth). | -Two years after previous rupture.  -Back pains (38 weeks); immediate CS: partial rupture in uterine fundus. | Alive and healthy. |
| Case 3 (1978-1988) | Para 2  -Emergency CS with vertical incision (28 weeks).  -Complete rupture in vertical scar (32 weeks) after labor (stillbirth). | -Five years after previous rupture.  -Irregular contractions (28 weeks); immediate CS: partial rupture in vertical scar. | Alive infant without asphyxia; admitted to NICU due to prematurity. |
| Case 4  (2000-2011) | Para 4  -Two uncomplicated vaginal deliveries.  -Emergency CS (29 weeks) with inverted T.  -Complete pre-labour rupture in inverted T scar (38 weeks; stillbirth). | -One year after previous rupture.  -Moderate abdominal pains similar to contractions (28 weeks); immediate CS: partial rupture in inverted T scar. | Alive infant without asphyxia; admitted to NICU due to prematurity. |
| Case 5 (2000-2011) | Para 2  -Elective LSCS (term).  -Complete rupture in uterotomy scar after labor (39 weeks; neonatal death). | -Two years after previous rupture.  -Irregular contractions (37 weeks); immediate CS: partial rupture in uterotomy scar. | Alive and healthy. |
| Case 6 (2000-2011) | Para 2  -Elective LSCS (35 weeks) due to intrauterine growth restriction.  - Complete rupture in uterotomy scar after induced labour (39 weeks). | -One year after previous rupture.  -Elective CS at 37 weeks: dehiscence in uterotomy scar. | Alive and healthy. |
